# Mechanically activated Piezo1 channels of cardiac fibroblasts stimulate p38 mitogen-activated protein kinase activity and interleukin-6 secretion

**DOI:** 10.1074/jbc.RA119.009167

**Published:** 2019-10-04

**Authors:** Nicola M. Blythe, Katsuhiko Muraki, Melanie J. Ludlow, Vasili Stylianidis, Hamish T. J. Gilbert, Elizabeth L. Evans, Kevin Cuthbertson, Richard Foster, Joe Swift, Jing Li, Mark J. Drinkhill, Frans A. van Nieuwenhoven, Karen E. Porter, David J. Beech, Neil A. Turner

**Affiliations:** ‡ Discovery and Translational Science Department, Leeds Institute of Cardiovascular and Metabolic Medicine, School of Medicine, University of Leeds, Leeds LS2 9JT, United Kingdom; § Multidisciplinary Cardiovascular Research Centre, University of Leeds, Leeds LS2 9JT, United Kingdom; ¶ Laboratory of Cellular Pharmacology, School of Pharmacy, Aichi-Gakuin University, 1–100 Kusumoto, Chikusa, Nagoya 464-8650, Japan; ‖ Department of Physiology, Cardiovascular Research Institute Maastricht, Maastricht University, Maastricht 6200MD, The Netherlands; ** Wellcome Centre for Cell-Matrix Research, Division of Cell Matrix Biology and Regenerative Medicine, School of[c27c]áBiological Sciences, Faculty of Biology, Medicine and Health, Manchester Academic Health Science Centre, University of Manchester, Manchester,[c27c]áM13 9PL, United Kingdom; ‡‡ School of Chemistry, University of Leeds, Leeds LS2 9JT, United Kingdom

**Keywords:** ion channel, calcium, p38 MAPK, mitogen-activated protein kinase (MAPK), IL-6, fibroblast, heart, mechanotransduction, patch clamp, signal transduction, cardiac fibroblast

## Abstract

Piezo1 is a mechanosensitive cation channel with widespread physiological importance; however, its role in the heart is poorly understood. Cardiac fibroblasts help preserve myocardial integrity and play a key role in regulating its repair and remodeling following stress or injury. Here we investigated Piezo1 expression and function in cultured human and mouse cardiac fibroblasts. RT-PCR experiments confirmed that *Piezo1* mRNA in cardiac fibroblasts is expressed at levels similar to those in endothelial cells. The results of a Fura-2 intracellular Ca^2+^ assay validated Piezo1 as a functional ion channel that is activated by its agonist, Yoda1. Yoda1-induced Ca^2+^ entry was inhibited by Piezo1 blockers (gadolinium and ruthenium red) and was reduced proportionally by siRNA-mediated Piezo1 knockdown or in murine *Piezo1*^+/−^ cells. Results from cell-attached patch clamp recordings on human cardiac fibroblasts established that they contain mechanically activated ion channels and that their pressure responses are reduced by Piezo1 knockdown. Investigation of Yoda1 effects on selected remodeling genes indicated that Piezo1 activation increases both mRNA levels and protein secretion of IL-6, a pro-hypertrophic and profibrotic cytokine, in a Piezo1-dependent manner. Moreover, Piezo1 knockdown reduced basal IL-6 expression from cells cultured on softer collagen-coated substrates. Multiplex kinase activity profiling combined with kinase inhibitor experiments and phosphospecific immunoblotting established that Piezo1 activation stimulates IL-6 secretion via the p38 mitogen-activated protein kinase downstream of Ca^2+^ entry. In summary, cardiac fibroblasts express mechanically activated Piezo1 channels coupled to secretion of the paracrine signaling molecule IL-6. Piezo1 may therefore be important in regulating cardiac remodeling.

## Introduction

Cardiac fibroblasts play important roles in the normal physiology of the heart and in its response to damage or stress. Altered environmental stiffness and mechanical stretching of cardiac fibroblasts, which occur secondary to fibrosis, cardiac dilatation, or changes in the hemodynamic burden in the heart, induce differentiation of fibroblasts into myofibroblasts with increased expression of pro-fibrotic cytokines, extracellular matrix (ECM)^5^ proteins, and ECM receptors (1–3). This preserves cardiac integrity and performance, but the response can become maladaptive; surplus deposition of cardiac ECM results in fibrosis, which increases the stiffness of the myocardium and reduces pumping capacity (4).

Much remains unknown regarding how mechanical forces are translated into transcriptional responses important for determination of the fibroblast phenotype and how this can lead to cardiac remodeling (3, 5). Stretch-activated ion channels have been identified as candidates for cardiac mechanotransducers (6). Intracellular Ca^2+^ controls many functions in cardiac fibroblasts, including ECM synthesis and cell proliferation (7, 8), and it is known that Ca^2+^ entry through ion channels is important for cardiac fibroblast responsiveness. For example, TRPV4 channel–mediated Ca^2+^ signaling has been shown to play a role in mechanosensation and differentiation of fibroblasts into myofibroblasts (9).

In 2010, the Piezo1 and Piezo2 proteins were identified as the long-sought molecular carriers of an excitatory mechanically activated current found in many cell types (10). Piezo1, a nonselective cation channel, can be activated by a synthetic small molecule called Yoda1 (11). Yoda1 was discovered in a screen of ∼3.25 million compounds aiming to identify activators of Piezo1 or Piezo2 using high-throughput Ca^2+^ imaging (11). Importantly, Yoda1 activates Piezo1 but not Piezo2 and can do so in artificial lipid bilayers without the need for other cellular components (11). More recently, Yoda1 has been used to explore the gating mechanism of Piezo1 (12). Hence, Yoda1 is a useful tool for activating the Piezo1 channel without the need for mechanical stimulation. *Piezo1* mRNA has been detected in the murine heart, whereas *Piezo2* mRNA was barely detected (10). Piezo1 has been shown recently to be expressed in cardiomyocytes and up-regulated in heart failure (13). However, there are currently no reports regarding the role of Piezo1 in cardiac fibroblasts. We hypothesized that Piezo1 plays an important role in cardiac fibroblast function by regulating Ca^2+^ entry and downstream signaling.

Our data provide evidence that Piezo1 acts as a functional Ca^2+^-permeable mechanosensitive ion channel in murine and human cardiac fibroblasts and that its activation by Yoda1 is coupled to secretion of IL-6, a cytokine important in the response to cardiac injury and hypertrophic remodeling. We further reveal that Piezo1-induced Ca^2+^ entry is coupled to IL-6 expression via activation of p38 MAPK.

## Results

### Piezo1 expression and activity in cardiac fibroblasts

**Figure 1.**
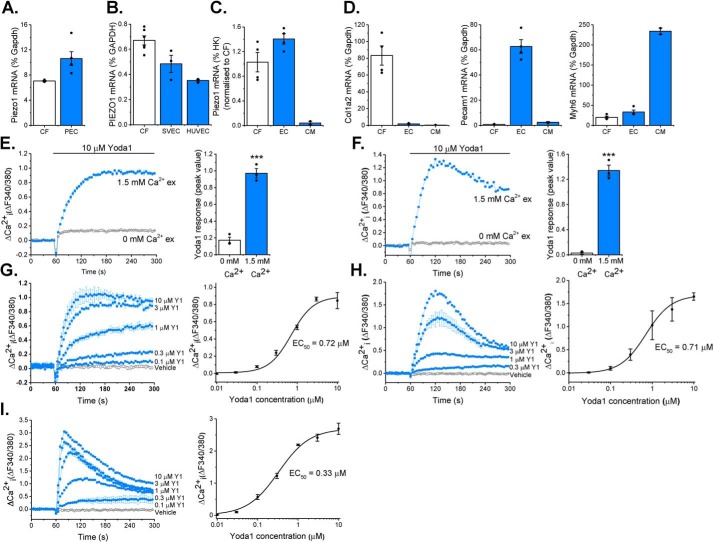
**Piezo1 is expressed by cardiac fibroblasts and forms a functional ion channel.** *A* and *B*, RT-PCR analysis of *Piezo1* mRNA expression in murine cardiac fibroblasts (CF, *n* = 3) compared with murine pulmonary endothelial cells (*PEC*, *n* = 5) (*A*) and human CF (*n* = 6) compared with human saphenous vein endothelial cells (*SVEC*, *n* = 3) and HUVECs (*n* = 3) (*B*). Expression was measured as percent of the housekeeping control (*Gapdh*/*GAPDH*). *C*, RT-PCR analysis of *Piezo1* mRNA expression in fibroblast-enriched fraction 2 (CF) and endothelial cell–enriched fraction 1 (*EC*) isolated from murine heart using the MACS technique (*n* = 4). Cardiomyocytes (*CM*) were isolated from separate hearts (*n* = 2). Expression was measured relative to three housekeeping genes (*Gapdh*, *Actb*, and *Hprt*) and normalized to the CF sample. *D*, RT-PCR analysis of cell type-specific marker genes for CF (*Col1a2*), endothelial cells (*Pecam1*), and cardiomyocytes (*Myh6*) in MACS fractions as for *C. E* and *F*, representative Ca^2+^ traces and mean ± S.E. are shown. Ca^2+^ entry was evoked by 10 μm Yoda1 in murine (*E*) and human (*F*) cardiac fibroblasts in the presence or absence of extracellular Ca^2+^. ***, *p* < 0.001 (paired *t* test; *n*/*N* = 3/9). *G–I*, Ca^2+^ entry evoked by varying concentrations of Yoda1 application at 60 s, ranging from 0.1–10 μm in murine (*G*) and human (*H*) cardiac fibroblasts and HEK T-Rex-293 cells heterologously expressing mouse Piezo1 (*I*). Vehicle control is illustrated by the *black trace*. Mean ± S.E. are displayed as concentration–response curves, and fitted curves are plotted using a Hill equation, indicating the EC_50_ of Yoda1 (*n*/*N* = 3/9).

mRNA encoding Piezo1 was detected in cultured cardiac fibroblasts from both mouse and human hearts (Fig. 1, *A* and *B*). In comparison with endothelial cells, which are known to express high levels of this channel (14), *Piezo1* mRNA expression levels in murine cardiac fibroblasts were similar to those observed in murine pulmonary endothelial cells, and those in human cardiac fibroblasts were similar to those in human saphenous vein endothelial cells and human umbilical vein endothelial cells (HUVECs) (Fig. 1, *A* and *B*). Using a magnetic antibody cell separation (MACS) technique (15), we confirmed that Piezo1 was expressed in the fibroblast-enriched (*Col1a2*-positive) fraction of freshly isolated cells from mouse heart at a level similar to that in the endothelial cell-enriched (*Pecam-1*-positive) fraction (Fig. 1, *C* and *D*). *Piezo1* mRNA levels were 20 times higher in isolated cardiac fibroblasts than *Myh6*-positive cardiomyocytes when normalized to the average of three separate housekeeping genes (Fig. 1, *C* and *D*).

Having demonstrated that cardiac fibroblasts express *Piezo1* mRNA, we investigated whether the Piezo1 protein was able to form a functional ion channel. Using the Fura-2 Ca^2+^ indicator assay, it was found that Yoda1, a Piezo1 agonist (11), elicited an increase in intracellular Ca^2+^ in murine and human cardiac fibroblasts (Fig. 1, *E* and *F*). Consistent with the Yoda1-induced increase in intracellular Ca^2+^ being due to influx of extracellular Ca^2+^ through an ion channel, the Ca^2+^ signal was reduced by more than 90% when extracellular Ca^2+^ was absent in human and mouse cardiac fibroblast cultures (Fig. 1, *E* and *F*).

Concentration–response data for Yoda1 in murine cardiac fibroblasts revealed a marked effect at 0.3 μm, and the maximal response was generated at 10 μm; the EC_50_ of Yoda1 was estimated to be 0.72 μm (Fig. 1*G*). This was almost identical to the EC_50_ observed in human cardiac fibroblasts in similar experiments (Fig. 1*H*) and comparable with that for mouse Piezo1 heterologously expressed in HEK T-REx^TM^-293 cells, where the EC_50_ of Yoda1 was 0.33 μm (Fig. 1*I*).

**Figure 2.**
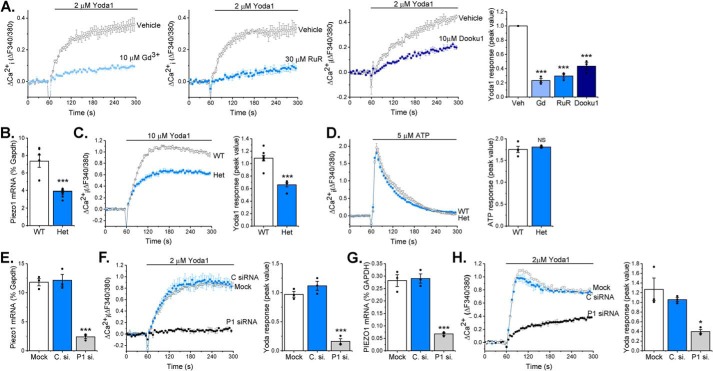
**Yoda1-evoked Ca^2+^ entry depends on Piezo1 expression.** *A*, representative intracellular Ca^2+^ traces and mean data after murine cardiac fibroblasts were exposed to 10 μm Gd^3+^, 30 μm ruthenium red (*RuR*), 10 μm Dooku1, or vehicle (*Veh*) for 30 min before activation of Piezo1 by application of 2 μm Yoda1. Data were normalized to vehicle-treated cells. Repeated measures one-way ANOVA: *p* < 0.0001, F = 114.1 (*n*/*N* = 3/9). Post hoc test: ***, *p* < 0.001 *versus* vehicle-treated cells. *B*, RT-PCR analysis of *Piezo1* mRNA expression in cultured murine cardiac fibroblasts isolated from WT and *Piezo1*^+/−^ (*Het*) mice. Expression is measured as percent of the housekeeping control *Gapdh*. ***, *p* < 0.001 (unpaired *t* test, *n* = 8). *C*, representative Ca^2+^ trace and mean data illustrating Ca^2+^ entry elicited by 10 μm Yoda in cardiac fibroblasts from WT (*n*/*N* = 8/24) and *Piezo1*^+/−^ Het (*n*/*N* = 5/15) mice. ***, *p* < 0.001 (unpaired *t* test). *D*, representative Ca^2+^ trace and mean data illustrating Ca^2+^ entry evoked by 5 μm ATP in cardiac fibroblasts from WT (*n*/*N* = 4/12) and *Piezo1*^+/−^ (*Het*, *n*/*N* = 3/9) mice. Unpaired *t* test: not significant (*NS*). *E*, RT-PCR analysis of *Piezo1* mRNA expression following transfection of murine cardiac fibroblasts with Piezo1 siRNA, mock-transfected cells, and cells transfected with control siRNA. Expression is measured as percent of the housekeeping control *Gapdh*. Repeated measures one-way ANOVA: *p* = 0.0001, F = 61.1 (*n* = 3). Post hoc test: ***, *p* < 0.001 *versus* mock-transfected cells. *F*, representative Ca^2+^ trace and mean data showing the response to 2 μm Yoda1 in murine cardiac fibroblasts transfected with Piezo1-specific siRNA (*P1 si.*) compared with mock-transfected cells and cells transfected with control siRNA (*C. si.*). Repeated measures one-way ANOVA: *p* < 0.0001, F = 72.6 (*n*/*N* = 3/9). Post hoc test: ***, *p* < 0.001 *versus* mock-transfected cells. *G*, as for *E* but in human cardiac fibroblasts. Repeated measures one-way ANOVA: *p* = 0.0002, F = 50.8 (*n*/*N* = 3/9). Post hoc test: ***, *p* < 0.001 *versus* mock-transfected cells. *H*, as for *F* but in human cardiac fibroblasts. Repeated measures one-way ANOVA: *p* = 0.0108, F = 10.6 (*n*/*N* = 3/9). Post hoc test: *, *p* < 0.05 *versus* mock-transfected cells.

Gadolinium (Gd^3+^) and ruthenium red, both nonspecific inhibitors of mechanosensitive ion channels, including Piezo1 (10), were used to investigate the pharmacology of the channel. Murine cardiac fibroblasts preincubated with these inhibitors exhibited significantly reduced Yoda1-evoked Ca^2+^ entry (>70% reduction in both cases) (Fig. 2*A*). Additionally, Yoda1-evoked Ca^2+^ entry was significantly reduced by more than 50% by Dooku1 (Fig. 2*A*), an analog of Yoda1 that has antagonist properties against Yoda1 in Piezo1-overexpressing HEK293 cells and HUVECs (16). The data were similar in human cardiac fibroblasts, where again all three inhibitors significantly reduced Ca^2+^ influx in response to Yoda1 (Fig. S1). Ca^2+^ entry elicited by Yoda1 application was also investigated in cardiac fibroblasts isolated from a global heterozygous (Het) *Piezo1*^+/−^ mouse line. RT-PCR analysis confirmed the predicted 50% reduction in *Piezo1* mRNA expression in cardiac fibroblasts derived from *Piezo1*^+/−^ hearts compared with the WT (Fig. 2*B*). The Ca^2+^ response to Yoda1 was reduced by 40% (Fig. 2*C*), whereas the response to ATP was similar in cells from WT and *Piezo1*^+/−^ mice (Fig. 2*D*). Piezo1-specific siRNA, which decreased *Piezo1* mRNA expression by 80% in murine cardiac fibroblasts (Fig. 2*E*), reduced Yoda1-evoked Ca^2+^ entry by a similar level (Fig. 2*F*), whereas control siRNA was without effect. Similar results were obtained with human cardiac fibroblasts (Fig. 2, *G* and *H*). Thus, the Yoda1 response depended on Piezo1 and was proportional to its expression level. Together, these data establish that Yoda1-induced Ca^2+^ entry in cardiac fibroblasts depends on Piezo1 expression and that the channel has the expected pharmacological properties.

### Cardiac fibroblasts contain mechanically activated currents

**Figure 3.**
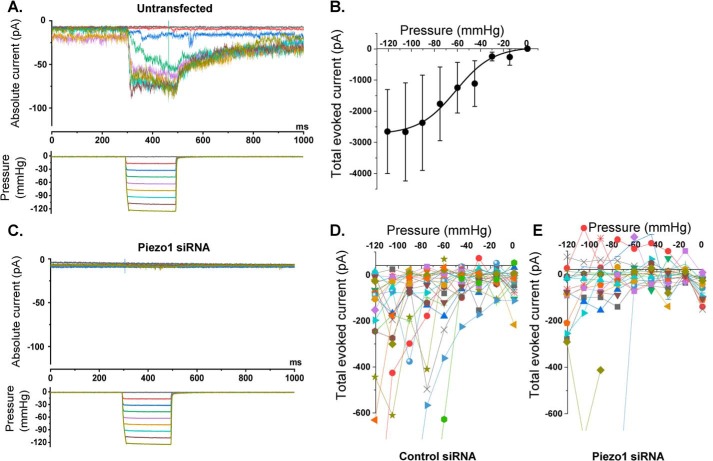
**Cardiac fibroblasts express mechanically activated ionic currents that depend on Piezo1.** Recordings were made from cell-attached patches on human cardiac fibroblasts. A constant holding voltage of +80 mV was applied to the patch pipette to ensure a negative membrane potential across the patch (inside relative to outside). A rapid pressure clamp system applied 200-ms negative pressure (suction) steps of increasing magnitude to the patch pipette. *A*, example data from an untransfected cell. The color code of the pressure steps (*bottom panel*) matches the color code of the current traces in the *top panel. B*, mean ± S.E. data for experiments of the type shown in *A*, in which the total current during 180 ms of each pressure pulse was summed for *n* = 7–8 patches/data point. The fitted curve is the Boltzmann function, which gave a midpoint for 50% activation of −61.3 mm Hg. *C*, as for *A* but from a cell that was transfected with Piezo1 siRNA. *D* and *E*, each color shows the individual data for all recordings from cells transfected with control siRNA (*D*, *n* = 24 recordings) or Piezo1 siRNA (*E*, *n* = 21 recordings). As for *B*, the total current during 180 ms of each pressure pulse was summed. Two-way ANOVA: *p* = 0.0475, F = 3.95 (siRNA); *p* < 0.0001, F = 4.15 (pressure); *p* < 0.0001, F = 4.16 (interaction). In *D* and *E*, data values exceeding approximately −600 pA are clipped to maximize visibility of the majority of the data, but all data values were included in the statistical analysis. The two large current values in the Piezo1 siRNA group may have been from nontransfected cells because the transfection efficiency was estimated to be 90%.

To investigate whether cardiac fibroblasts contain mechanically activated ion channels, we made cell-attached patch recordings from human cardiac fibroblasts. Mechanical force was applied to the patches using a fast pressure clamp system that generated calibrated suction pulses (pressure pulses) in the patch pipette and therefore increased membrane tension (Fig. 3*A*). Increased pressure caused increased inward currents up to a limit (Fig. 3, *A* and *B*). The currents were noisy macroscopic currents, suggesting the presence of multiple individual channels that summed to generate the overall current. The amplitude of the current was highly variable between patches, as seen by the large standard error in Fig. 3*B*. Despite this variability, we estimated that the pressure required for 50% activation was −61.3 mm Hg (Fig. 3*B*).

### The mechanically activated channel activity is Piezo1-dependent

To test whether the currents were Piezo1-dependent, we systematically compared two matched groups of human cardiac fibroblasts: one transfected with control siRNA and the other transfected with Piezo1 siRNA. Because the transfection efficiency was estimated to be 90% at best, we expected that at least one in every 10 recordings would be from a nontransfected cell. To compensate for this limitation, we made a large number of individual recordings before analyzing the data (45 in total). Our overall impression was that most Piezo1 siRNA–transfected cells showed no meaningful current in response to pressure steps (Fig. 3*C*). Data for all 45 recordings are shown in Fig. 3, *D* and *E*. The high variability was again observed, but it was also visually apparent that pressure-activated currents were more common in the control siRNA group than in the Piezo1 siRNA group (compare Fig. 3, *D* and *E*). In only two patches of the Piezo1 siRNA group were clear pressure-activated currents observed (Fig. 3*E*). We suspected that these latter two recordings were from nontransfected cells (assuming 90% transfection efficiency). Formal analysis using two-way ANOVA on all data in the two groups for all pressure steps (no data were excluded) indicated that currents in the Piezo1 siRNA group were statistically significantly different (smaller) than those in the control siRNA group. These data suggest that human cardiac fibroblasts express mechanically activated Piezo1 channels.

### Yoda1-induced Piezo1 activation is coupled to increased Il6 gene expression

**Figure 4.**
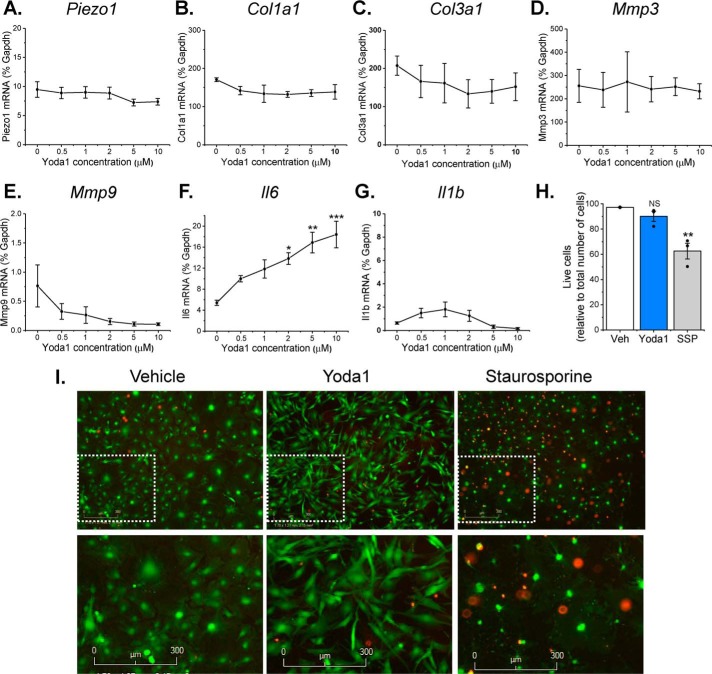
**Effect of Yoda1 on gene expression and cell survival in cardiac fibroblasts.** *A–G*, RT-PCR analysis of murine cardiac fibroblasts treated with concentrations of Yoda1 ranging from 0.5–10 μm or DMSO vehicle for 24 h (*n* = 3). All mRNA expression levels were normalized to those of the housekeeping gene *Gapdh*. Repeated measures one-way ANOVA: *p* = 0.0010, F = 8.9. Post hoc test: *, *p* < 0.05; **, *p* < 0.01; ***, *p* < 0.001 *versus* vehicle-treated cells. *H*, live/dead cell assay performed on cultured murine cardiac fibroblasts treated with either vehicle (*Veh*), 10 μm Yoda1, or 1 μm staurosporine (*SSP*) for 24 h. The bar chart shows mean data for viable cells as a percentage of total cells. Repeated measures one-way ANOVA: *p* = 0.0028, F = 10.6 (*n* = 3). Post hoc test: **, *p* < 0.01; *NS*, not significant *versus* vehicle-treated cells. *I*, representative images from the live/dead cell assay. *Green* indicates live cells; *red* indicates dead cells. *Bottom panels* are magnified images of the indicated regions in the *top panels*.

To gain insight into the functional role of Piezo1 activation in cardiac fibroblasts, the effect of 24-h treatment with 0.5–10 μm Yoda1 on the expression of selected remodeling genes in murine fibroblasts was investigated by RT-PCR (Fig. 4, *A–G*). Prolonged Yoda1 treatment did not modulate *Piezo1* gene expression (Fig. 4*A*), nor did it affect the expression of genes involved in ECM turnover, including collagens I and III (*Col1a1*, *Col3a1*) or matrix metalloproteinase 3 and 9 (*Mmp3*, *Mmp9*) (Fig. 4, *B–E*). However, a concentration-dependent increase in mRNA expression of the inflammatory/hypertrophic cytokine IL-6 was observed, with significant 2- to 4-fold increases observed in response to 2–10 μm Yoda1 (Fig. 4*F*). This was not a generic inflammatory response because *Il1b* mRNA levels remained unaffected by Yoda1 treatment (Fig. 4*G*). Prolonged exposure to Yoda1 did not affect cell viability (Fig. 4, *H* and *I*), although it did appear to induce a morphological change in the cells toward a more spindle-like, less rhomboid shape (Fig. 4*I*).

### Piezo1 activation stimulates IL-6 expression and secretion via p38 MAP kinase

**Figure 5.**
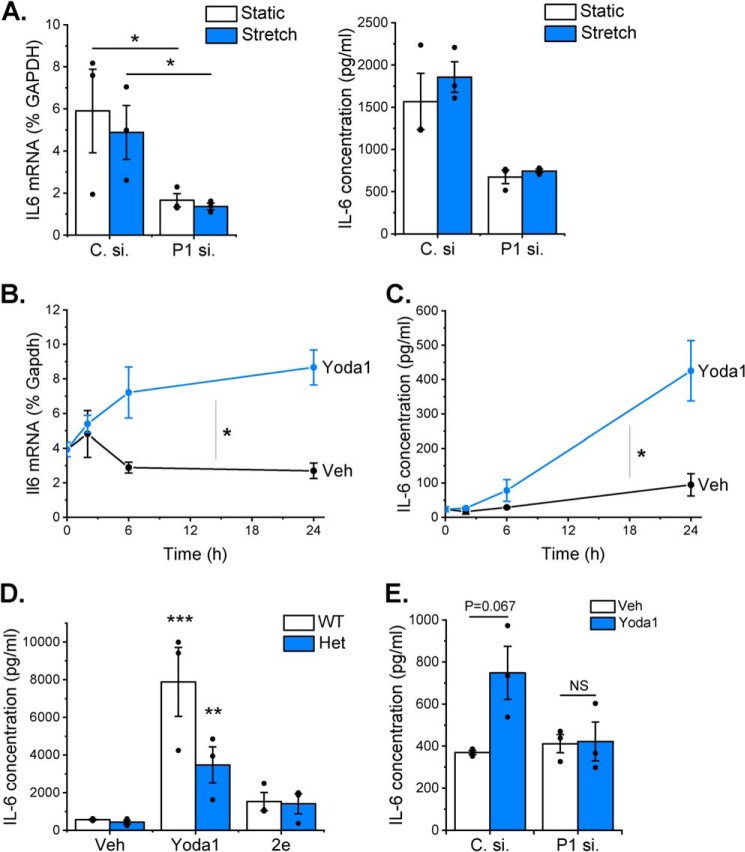
**Activation of Piezo1 is coupled to IL-6 expression.** *A*, RT-PCR analysis of *IL6* mRNA expression (*left panel*) and ELISA analysis of IL-6 protein secretion (*right panel*) after exposure of human cardiac fibroblasts to 24-h cyclical stretch (1 Hz, 10% stretch) on collagen-coated BioFlex plates compared with fibroblasts maintained in parallel under static conditions. Cardiac fibroblasts were transfected previously with either control siRNA (*C. si.*) or Piezo1-specific siRNA (*P1 si.*). RT-PCR data are expressed as percent of the housekeeping control *GAPDH*. Repeated measures two-way ANOVA for RT-PCR (*n* = 3): *p* = 0.0704, F = 12.7 (siRNA); *p* = 0.403, F = 1.1 (stretch); *p* = 0.754, F = 0.13 (interaction). Post hoc test: *, *p* < 0.05. Repeated measures two-way ANOVA for ELISA (*n* = 3): *p* = 0.0268, F = 35.8 (siRNA); *p* = 0.1545, F = 5.0 (stretch); *p* = 0.838, F = 0.05 (interaction). Post hoc test: not statistically significant. *B* and *C*, murine cardiac fibroblasts were exposed to vehicle (*Veh*) or 10 μm Yoda1 for 2–24 h before measuring *Il6* mRNA levels by RT-PCR, where expression is measured as percent of the housekeeping control *Gapdh* (*B*) or analyzing conditioned medium for IL-6 levels by ELISA (*C*) (both *n* = 3). Comparison of the area under the curve by paired *t* test: *, *p* < 0.05 for the effect of Yoda1 on IL-6 expression at both mRNA and protein levels. *D*, cardiac fibroblasts from WT (*n* = 4) and *Piezo1*^+/−^ (*Het*, *n* = 3) mice were treated with vehicle, 10 μm Yoda1, or compound 2e for 24 h before measuring IL-6 levels in conditioned medium using ELISA. Two-way ANOVA: *p* < 0.0001, F = 24.9 (compound); *p* = 0.1041, F = 3.1 (genotype); *p* = 0.615, F = 0.51 (interaction). Post hoc test: ***, *p* < 0.001; **, *p* < 0.01 for the effect of Yoda1 *versus* vehicle. All other changes were not statistically significant. *E*, mouse cardiac fibroblasts were transfected with control or Piezo1-specific siRNA and treated with either vehicle or 10 μm Yoda1 for 24 h before collecting conditioned media and measuring IL-6 levels by ELISA (*n* = 3). Repeated measures two-way ANOVA: *p* = 0.2349, F = 2.82 (siRNA); *p* = 0.2187, F = 3.13 (Yoda1); *p* = 0.064, F = 14.2 (interaction). Post hoc test: *NS*, not significant *versus* vehicle-treated cells.

Given our recent findings regarding the potential importance of cardiac fibroblast-derived IL-6 in modulating cardiac hypertrophy (15) and the causative link between mechanical stimulation and cardiac hypertrophy (17), we proceeded to interrogate the mechanism by which Piezo1 activation was coupled to IL-6 expression. First, we explored whether mechanical activation of cardiac fibroblasts was coupled to IL-6 expression and whether this occurred via a Piezo1-dependent mechanism. Cyclic stretching (1 Hz, 10% stretch) of human cardiac fibroblasts for 24 h had no effect on *IL6* mRNA levels or IL-6 protein secretion (Fig. 5*A*). However, during these experiments, it became apparent that *PIEZO1* gene silencing significantly reduced basal IL-6 expression at both mRNA and protein levels (independently of stretch) when cells were grown on BioFlex plates (Fig. 5*A*) but not when they were maintained on regular tissue culture plastic (Fig. 5*E*). The BioFlex plates have a stiffness (Young's modulus) ∼1000 times less than standard tissue culture plates and were coated with type I collagen. Thus, activation of Piezo1 by chemical activation (Yoda1) or in response to altered substrate composition/stiffness was coupled to increased IL-6 expression in cardiac fibroblasts.

We then probed the mechanism further in murine cardiac fibroblasts using Yoda1 as a stimulus. *Il6* mRNA expression increased in a time-dependent manner over a 2- to 24-h period of Yoda1 treatment (Fig. 5*B*). This increase correlated with an increase in IL-6 protein secretion (Fig. 5*C*). Yoda1-induced IL-6 secretion was reduced by ∼50% in cardiac fibroblasts isolated from hearts of *Piezo1*^+/−^ mice compared with those from WT hearts (Fig. 5*D*), in keeping with the reduction in Piezo1 expression and Ca^2+^ response in these cells (Fig. 2, *B* and *C*). IL-6 secretion was unchanged following treatment of cardiac fibroblasts with compound 2e (Fig. 5*D*), an inactive analogue of Yoda1 (16) that did not induce Ca^2+^ entry in murine or human cardiac fibroblasts or in HEK T-REx-293 cells heterologously expressing mouse Piezo1 (Fig. S2, *A–C*). The Yoda1-induced increase in IL-6 secretion depended on Piezo1, as evidenced by attenuation of the secretion in murine cardiac fibroblasts transfected with Piezo1-specific siRNA (Fig. 5*E*). The same was true in human cardiac fibroblasts, where the Yoda1-evoked increase in *Il6* mRNA expression was attenuated by Piezo1-specific siRNA, and again the inactive Yoda1 analog 2e had no effect (Fig. S2, *D* and *E*). These data establish that Yoda1 induces IL-6 expression in cardiac fibroblasts via a Piezo1-dependent mechanism.

**Figure 6.**
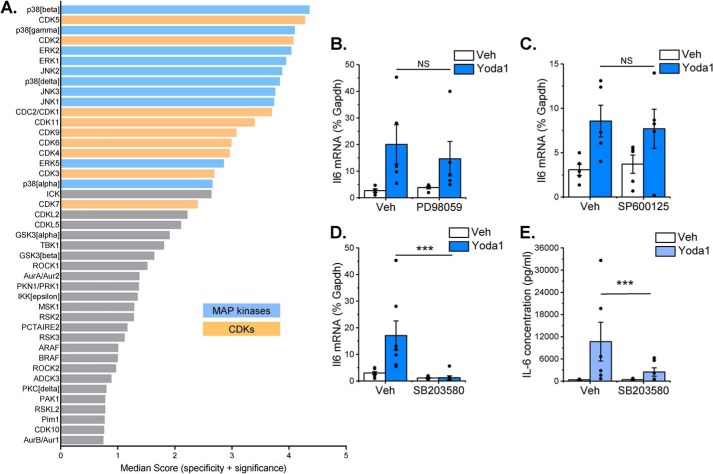
**Piezo1 activation induces MAP kinase signaling, with p38 MAPK being coupled to IL-6 expression.** A, mouse cardiac fibroblasts were stimulated with 10 μm Yoda1 for 10 min before analyzing Ser/Thr protein kinase activity with PamChip multiplex kinase activity profiling (*n* = 6). Kinases were identified based on peptide substrate phosphorylation and ranked by specificity and statistical significance (only the top 40 are shown). See Table S1 for the full dataset. The top kinase families predicted to be activated by Yoda1 were the MAPKs ERK1/2/5, JNK1/2/3, and p38α/β/γ/δ (*blue*) and CDKs 1–7, 9, and 11 (*orange*). *B–D*, RT-PCR analysis of *Il6* mRNA expression after exposure of murine cardiac fibroblasts to 30 μm PD98059 (*B*, an ERK pathway inhibitor), 10 μm SP600125 (*C*, a JNK inhibitor), or 10 μm SB203580 (*D*, a p38 MAPK inhibitor) for 1 h, followed by treatment with vehicle (*Veh*) or 10 μm Yoda1 for a further 24 h. Expression is measured as percent of the housekeeping control *Gapdh* (*n* = 7 for SB203580, *n* = 5 for PD98059, and SP600125). Repeated measures two-way ANOVA for *B*: *p* = 0.7738, F = 0.133 (PD98059); *p* = 0.0529, F = 7.41 (Yoda1); *p* = 0.069, F = 6.08 (interaction). For *C*: *p* = 0.6133, F = 0.299 (SP600125); *p* = 0.0020, F = 51.9 (Yoda1); *p* = 0.475, F = 0.62 (interaction). For *D*: *p* = 0.0004, F = 52.1 (SB203580); *p* = 0.2942, F = 1.32 (Yoda1); *p* = 0.0028, F = 23.8 (interaction). Post hoc test: ***, *p* < 0.001; *NS*, not significant for the effect of inhibitor. *E*, murine cardiac fibroblasts were treated with vehicle or 10 μm SB203580 for 1 h and then treated with either vehicle or 10 μm Yoda1 for 24 h before collecting conditioned media and measuring IL-6 levels by ELISA (*n* = 6). Repeated measures two-way ANOVA: *p* = 0.0217, F = 10.8 (SB203580); *p* = 0.0283, F = 9.3 (Yoda1); *p* = 0.0002, F = 98.1 (interaction). Post hoc test: ***, *p* < 0.001 for the effect of SB203580.

PamChip multiplex serine/threonine kinase activity profiling was used to assess differences in kinase activity following treatment of murine cardiac fibroblasts with Yoda1 for 10 min. Combinatorial analysis of phosphorylation of 140 peptide substrates revealed a hierarchical list of predicted serine/threonine kinases that were activated downstream of Piezo1 (Fig. 6*A* and Table S1). Within the top 20 hits, two major kinase families were identified: MAPKs (extracellular signal-regulated kinases (ERK1/2/5), c-Jun N-terminal kinases (JNK1/2/3), and p38 mitogen-activated protein kinases (p38α/β/γ/δ)) and cyclin-dependent kinases (CDK 1–7, 9, and 11) (Fig. 6*A*). The primary role of CDK family kinases is to phosphorylate cell cycle proteins and thereby regulate cell cycle progression, although it has also been reported that specific CDKs can regulate inflammatory gene expression through interaction with the NF-κB pathway (18). However, given that kinases within the NF-κB pathway (IKKα, IKKβ) were not activated in response to Yoda1 in our experiments (Table S1), we did not pursue the role of CDKs further. Subsequent experiments were therefore undertaken with selective pharmacological inhibitors of the ERK (PD98059), JNK (SP600125), and p38 MAPK (SB203580) pathways to identify which were required for expression of *Il6* mRNA in response to Piezo1 activation in murine cardiac fibroblasts (Fig. 6, *B–D*). Of these, only SB203580 significantly reduced the Yoda1-induced increase in *Il6* mRNA levels back to basal levels (Fig. 6*D*), suggesting an important role for the p38 MAPK pathway in Yoda1-induced *Il6* gene expression. In agreement, SB203580 also inhibited Yoda1-induced IL-6 protein secretion by 80% (Fig. 6*E*).

**Figure 7.**
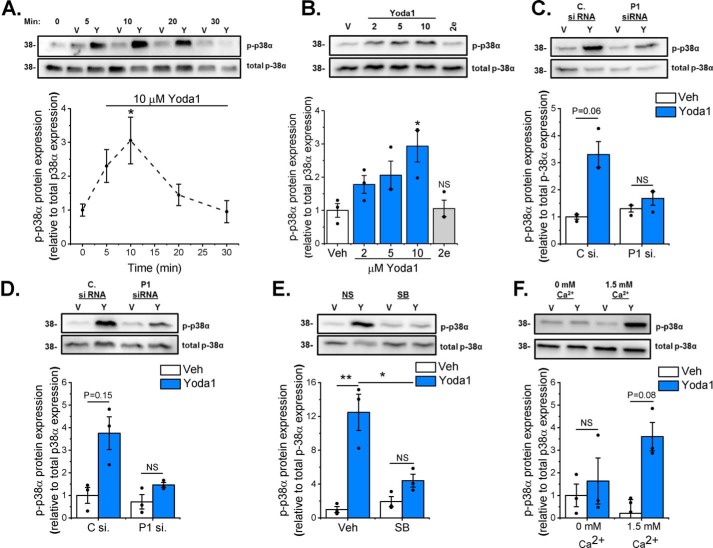
**Yoda1-induced p38 MAPK phosphorylation depends on Piezo1.** Cellular protein samples were immunoblotted for p-p38α and reprobed with antibody for total p38α to confirm equal protein loading. The bar charts show mean densitometric data of p-p38α normalized to p38α expression. *A*, murine cardiac fibroblasts (*n* = 6) treated with DMSO vehicle (*V*) or 10 μm Yoda1 (*Y*) for 5–30 min. *, *p* < 0.05 *versus* vehicle-treated cells (repeated measures one-way ANOVA, *p* = 0.0085). *B*, murine cardiac fibroblasts (*n* = 3) treated for 10 min with varying concentrations of Yoda1 (2–10 μm) or 10 μm compound 2e. *, *p* < 0.05; *NS*, not significant *versus* vehicle-treated cells (repeated measures one-way ANOVA, *p* = 0.0122). *C* and *D*, murine (*C*) or human (*D*) cardiac fibroblasts (*n* = 3) transfected with either scrambled or Piezo1-specific siRNA before treatment with vehicle or 10 μm Yoda1 for 10 min. Repeated measures two-way ANOVA for *C*: *p* = 0.0202, F = 48.0 (Yoda1); *p* = 0.3045, F = 1.87 (siRNA); *p* = 0.0903, F = 9.6 (interaction). Repeated measures two-way ANOVA for *D*: *p* = 0.0282, F = 34.0 (Yoda1); *p* = 0.2669, F = 2.32 (siRNA); *p* = 0.478, F = 0.75 (interaction). Post hoc test: not significant. *E*, murine cardiac fibroblasts (*n* = 3) exposed to 10 μm SB203580 for 1 h before treatment with vehicle or 10 μm Yoda1 for 10 min. Repeated measures two-way ANOVA: *p* = 0.0312, F = 30.6 (Yoda1); *p* = 0.5898, F = 0.40 (SB203580); *p* = 0.0148, F = 65.9 (interaction). Post hoc test: **, *p* < 0.01; *, *p* < 0.05. *F*, murine cardiac fibroblasts (*n* = 3) treated for 10 min with vehicle or 10 μm Yoda1 in either standard DMEM or DMEM containing 1.75 mm EGTA to chelate free Ca^2+^. Repeated measures two-way ANOVA: *p* = 0.0123, F = 79.5 (Yoda1); *p* = 0.5739, F = 0.44 (Ca^2+^); *p* = 0.128, F = 6.34 (interaction). Post hoc test: not significant.

Western blotting was used to investigate whether the p38 MAPK pathway was stimulated following Piezo1 activation. Yoda1 increased p38 phosphorylation (activation) after 10 min before returning to basal levels after 30 min (Fig. 7*A*). Further studies investigating p38 activation revealed that Yoda1-induced p38 phosphorylation occurred in a concentration-dependent manner and that p38 was not activated in response to the inactive Yoda1 analog 2e (Fig. 7*B*). Piezo1-specific siRNA reduced Yoda1-induced activation of p38α in both murine (Fig. 7*C*) and human (Fig. 7*D*) cardiac fibroblasts, confirming the role of Piezo1 in Yoda1-induced p38 activation. The p38 inhibitor SB203580 significantly reduced the Yoda1-induced increase in p38 phosphorylation (Fig. 7*E*). Furthermore, p38 activation following Yoda1 treatment was shown to be dependent on the presence of extracellular Ca^2+^ (Fig. 7*F*), indicating that it is Ca^2+^ entry through the Piezo1 channel that is important for downstream signaling. Together, these data demonstrate that Ca^2+^-induced p38 MAPK activation is essential for inducing the expression and secretion of IL-6 in response to Piezo1 activation.

## Discussion

The three main findings of this study are that human and mouse cardiac fibroblasts express functional Piezo1 channels coupled to a rapid rise in intracellular Ca^2+^, that these channels are mechanosensitive, and that Piezo1 activation is coupled to secretion of IL-6 via a p38 MAPK-dependent pathway. These data establish a link between cardiac fibroblast Piezo1 and secretion of paracrine signaling molecules that can modulate cardiac remodeling.

We established that murine and human cardiac fibroblasts express *Piezo1* mRNA at levels similar to those found in endothelial cells from various sources, which are known to express high levels of functional Piezo1 (14). *Piezo1* mRNA expression was 20 times higher in cardiac fibroblasts than in cardiomyocytes. The EC_50_ values of Yoda1 acting on endogenous Piezo1 in murine and human cardiac fibroblasts (0.72 μm and 0.71 μm, respectively) were comparable with our previous report of 0.23 μm in HUVECs (16). When artificially overexpressed in cell lines, our data on mouse Piezo1 in HEK T-REx-293 cells (EC_50_ = 0.33 μm) were broadly comparable with human Piezo1 expressed in HEK T-REx-293 cells (EC_50_ = 2.51 μm) (16) and somewhat lower than those originally reported in HEK293T cells transiently transfected with mouse (EC_50_ = 17.1 μm) or human (EC_50_ = 26.6 μm) Piezo1 (11). It is worth noting that we could only use Yoda1 at concentrations up to 10 μm because of solubility problems, so these EC_50_ values are estimates.

The ability of common inhibitors of mechanosensitive ion channels, gadolinium and ruthenium red (10), and the novel Yoda1 antagonist Dooku1 (16) to decrease the Yoda1-evoked Ca^2+^ entry in murine and human cardiac fibroblasts correlated with the expected properties of the channel. The mechanism by which Yoda1 modulates Piezo1 activation and gating has been described recently (12). However, it has also been suggested that Yoda1 may have some Piezo1-independent effects in endothelial cells (19). We further verified the importance of Piezo1 by showing that the Ca^2+^, p38 MAPK, and IL-6 responses in cardiac fibroblasts were all proportionally reduced by siRNA-mediated knockdown of Piezo1 or by use of cells from a global *Piezo1*^+/−^ heterozygous knockout mouse model (14). Thus, the Yoda1 responses we observed in cardiac fibroblasts were unequivocally due to activation of Piezo1.

Our patch clamp data showed that increased pressure caused increased inward currents up to a limit, consistent with the presence of Piezo1 channels (10, 20). The amplitude of the current was highly variable between patches; we suspect that the variable amount of suction required to form the initial Giga-seal was a contributor to the variability in current amplitude (with greater suction apparently leading to smaller mechanically activated currents). Prior work has suggested that overexpressed human Piezo1 channels require −43 mm Hg for 50% activation (20), but the difference between this value and what we observed (−61.3 mm Hg) may reflect the influence of the native environment on channel properties at natural density. Although the kinetics of the currents were relatively slow and did not show inactivation, which contrasts with data for overexpressed Piezo1 channels (10), prior work has suggested that endogenous Piezo1 channels have slower kinetics and inactivate slowly or not at all (14, 21). These data suggest that Piezo1 channel activity most likely explains these mechanically activated currents in human cardiac fibroblasts.

Prolonged exposure to Yoda1 increased IL-6 mRNA and protein expression in a Piezo1-dependent manner in both human and mouse cardiac fibroblasts. IL-6 is a pleiotropic pro-inflammatory cytokine that promotes cardiac fibroblast proliferation and fibrosis (22) as well as cardiac hypertrophy through its actions on cardiomyocytes (23) and is readily secreted by cardiac fibroblasts in culture (24). Mechanical stretching of cardiac fibroblasts induces expression of several pro-fibrotic cytokines (1, 25), although IL-6 expression does not appear to be mechanically regulated in human cardiac fibroblasts (26), as we found in this study. This is in contrast to fibroblasts from various other noncardiac sources that have been shown to secrete IL-6 in response to mechanical stretch (27–29).

Although cyclic stretch did not modulate IL-6 expression in human cardiac fibroblasts (and no role for Piezo1 was found), it was apparent from the stretch experiments that Piezo1 siRNA could reduce basal IL-6 expression. This was evident only when cells were grown on the softer collagen-coated substrate of the BioFlex plates but not on the rigid plastic of regular tissue culture plates. Thus, Piezo1 may play a role in detecting altered substrate stiffness and/or composition and modulate IL-6 expression accordingly. It has been shown previously that stretch-induced expression of several cardiac fibroblast genes depends on matrix stiffness (3, 25); hence, there is a complex interplay of mechanosignaling pathways that convert these different mechanical stimuli into alterations in cellular phenotype. Further studies are required to more precisely determine the role of substrate stiffness in regulating IL-6 secretion in cardiac fibroblasts.

The p38 family of stress-activated MAPKs is known to play an important role in cardiac signaling and is activated in both acute and chronic cardiac pathologies (30). Recently, a key role for p38α in regulating multiple aspects of cardiac fibroblast function has emerged (31). For example, fibroblast-specific knockout of *Mapk14*, the gene encoding p38α, revealed a critical role for this kinase in driving cardiac myofibroblast differentiation and fibrosis in response to ischemic injury or chronic neurohumoral stimulation (32). In a similar model, we established a role for cardiac fibroblast p38α in stimulating cardiac hypertrophy after chronic β-adrenergic stimulation and uncovered a potential role for IL-6 acting as a paracrine inducer of cardiomyocyte hypertrophy in this context (15). We have also shown an important role for p38α in cytokine-induced IL-6 expression and secretion in cultured human cardiac fibroblasts (33). Thus, Piezo1-mediated activation of p38α in cardiac fibroblasts and subsequent secretion of IL-6 may be important in the cardiac remodeling process. Interestingly, hydrostatic pressure can stimulate p38 MAPK signaling through Piezo1 activation in mesenchymal stem cells (34).

In conclusion, our study establishes that Piezo1 is a functional mechanosensitive Ca^2+^-permeable ion channel in cardiac fibroblasts and is coupled to altered expression of genes important to the remodeling process. Specifically, activation of Piezo1 induces expression and secretion of IL-6, a pro-hypertrophic cytokine with important roles after cardiac injury. Furthermore, we revealed that p38 MAPK is activated downstream of Piezo1-mediated Ca^2+^ entry to cause increased IL-6 secretion. Functional studies and *in vivo* investigation into the role of Piezo1 in cardiac fibroblasts are warranted to provide additional information regarding its role in regulating cardiac remodeling.

## Experimental procedures

### Reagents

Yoda1 (Tocris), staurosporine (Sigma), PD98059 (Merck), SP600125 (Cambridge Bioscience), and SB203580 (Merck) were all solubilized in DMSO. ATP, gadolinium, and ruthenium red were all obtained from Sigma-Aldrich and dissolved in H_2_O. Dooku1 and compound 2e (16) were synthesized at the University of Leeds and solubilized in DMSO.

### Mouse cardiac fibroblast culture

Adult C57BL/6J mice were euthanized according to guidelines of The UK Animals (Scientific Procedures) Act (1986). Cardiac fibroblast cultures were established from collagenase-digested hearts and cultured in DMEM supplemented with 10% fetal calf serum as described previously (15). All cells were kept at 37 °C and 5% CO_2_ throughout the study. Cells were studied at passage 1–2. Cells were kept in serum-free DMEM for 16 h prior to treatment to collect RNA, lysates, and conditioned media.

### Human cell culture

Biopsies of human atrial appendages and long saphenous veins were obtained from patients undergoing elective coronary artery bypass grafting at the Leeds General Infirmary following local ethical committee approval and informed patient consent. The study complied with the principles of the Declaration of Helsinki. Cells were harvested and cultured as described previously for cardiac fibroblasts (35) and saphenous vein endothelial cells (36). Experiments were performed on human cardiac fibroblasts from passages 2–5. Serum-free DMEM was used for 1 h prior to treatment to collect RNA and conditioned media. HUVECs were purchased from Lonza and cultured in endothelial cell growth medium (EGM-2, Lonza), supplemented with 2% fetal calf serum and the EGM-2 SingleQuots Kit (Lonza). HUVECs were used at passage 2.

### Mouse pulmonary endothelial cell culture

CD146^+^ pulmonary endothelial cells were isolated with immunomagnetic microbeads (Miltenyi) and cultured in MV2 medium (Promocell) supplemented with 10% fetal calf serum, 100 units/ml penicillin, and 100 mg/ml streptomycin. Cells were studied at passage 1.

### HEK T-Rex-293 cell culture

pcDNA3_mouse Piezo1_IRES_GFP, a kind gift from Ardem Patapoutian (10), was used as a template to clone the mouse Piezo1 coding sequence into pcDNA4/TO. Overlapping mouse Piezo1 (forward primer 5′-GTAACAACTCCGCCCCATTG-3′ and reverse primer 5′-GCTTCTACTCCCTCTCACGTGTC-3′) and pcDNA4/TO (forward primer 5′-GACACGTGAGAGGGAGTAGAAGCCGCTGATCAGCCTCGACTG-3′ and reverse primer 5′-CAATGGGGCGGAGTTGTTAC-3′) PCR products were assembled using Gibson Assembly (New England Biolabs) (37). This construct does not contain tetracycline operator sequences. HEK T-REx-293 cells (Invitrogen) were transfected with pcDNA4/TO-mPiezo1 using Lipofectamine 2000 (Invitrogen) and treated with 200 μg/ml zeocin (InvivoGen) to select stably transfected cells. Individual clones were isolated and analyzed for expression using Yoda1 and intracellular Ca^2+^ measurements. HEK T-REx-293 cells were maintained in DMEM supplemented with 10% fetal calf serum and 1% penicillin/streptomycin (Sigma-Aldrich). Nontransfected HEK T-Rex-293 cells were used as control cells.

### Cardiac cell fractionation

Nonmyocyte cardiac cell fractions were prepared as described previously (15). Briefly, collagenase-digested heart tissue was filtered through a 30-μm MACS Smart Strainer (Miltenyi) to remove cardiomyocytes. Nonmyocytes were then separated into two fractions using a cardiac fibroblast magnetic antibody cell separation kit (Miltenyi). “Nonfibroblasts” (*Pecam1*-positive endothelial cells and leukocytes) were collected in fraction 1, and “fibroblasts” (*Col1a1/Col1a2/Ddr2/Pdgfra*-positive) were collected in fraction 2, as characterized previously (15). Separately, adult mouse cardiomyocytes were isolated from ventricles of 8-week-old WT mice. Hearts were cannulated through the aorta and perfused for 5 min with perfusion buffer (124.5 mm NaCl, 10 mm HEPES, 11.1 mm glucose, 1.2 mm NaH_2_PO_4_, 1.2 mm MgSO_4_, 4 mm KCl, and 25 mm taurine (pH 7.34)) containing 10 mm butanedione monoxime, followed by perfusion buffer containing 1 mg/ml type 2 collagenase, 0.05 mg/ml protease and 12.5 μm CaCl_2_ for 7–15 min until suitable digestion was observed. Ventricles were gently cut in perfusion solution containing 10% FBS and 12.5 μm CaCl_2_ and filtered. This step was repeated in perfusion solution containing 5% FBS and 12.5 μm CaCl_2_ and, finally, perfusion solution containing only 12.5 μm CaCl_2_. The filtrate was pelleted by gravity for 5 min before resuspension in perfusion buffer containing 0.5 mm CaCl_2_. This step was repeated with increasing concentrations of CaCl_2_ (1.5 mm, 3.5 mm, 8 mm, and 18 mm). Finally, the cell pellet was resuspended in 1 ml TRIzol. RNA was extracted from cardiac cell fractions, and quantitative RT-PCR was used to quantify gene expression.

### Quantitative RT-PCR

For gene expression studies, cardiac fibroblasts were treated with Yoda1 or vehicle (DMSO) for the indicated time. RNA was extracted from cultured/fractionated cells with the Aurum RNA Extraction Kit (Bio-Rad). cDNA was synthesized using a reverse transcription system (Promega). Real-time RT-PCR was performed with the ABI-7500 system, gene expression master mix, and specific TaqMan primer/probe sets (Thermo Fisher Scientific): mouse *Col1a1* (Mm01302043_g1), mouse *Col3a1* (Mm01254476_m1), mouse *Piezo1* (Mm01241545_g1), human *PIEZO1* (Hs00207230_m1), mouse *Il1b* (Mm00434228_m1), mouse *Il6* (Mm00446190_m1), human *IL6* (Hs00174131_m1), mouse *Mmp3* (Mm00440295_m1), and mouse *Mmp9* (Mm00442991_m1). Data are routinely expressed as the percentage of mouse *Gapdh* (Mm99999915_g1) or human *GAPDH* (Hs99999905_m1) housekeeping gene mRNA expression using the formula 2^−ΔCT^ × 100, in which C_T_ is the cycle threshold number. For analysis of cardiac cell fractions, three housekeeping genes were used to mitigate the effects of any variation in expression of housekeeping genes between cell fractions: *Gapdh* (Mm99999915_g1), *Hprt* (Mm03024075_m1), and *Actb* (Mm00607939_s1). Data were expressed relative to the geometric mean of the pooled housekeeping gene expression using the formula 2^−ΔCT^.

### Piezo1-modified mice

Animal use was authorized by the University of Leeds Animal Ethics Committee and by the UK Home Office under project license P144DD0D6. Animals were maintained in individually ventilated Optimice cages (Animal Care Systems) at 21 °C, 50–70% humidity, 12 h/12 h light/dark cycle, RM1 diet (Special Diet Services) *ad libitum*, and Pure'o Cell bedding (Datesand). C57BL/6 mice carrying global disruption of the *Piezo1* gene with a *lacZ* insertion flanked by Flp recombinase target sites have been described previously (14). Identification of WT and *Piezo1*^+/−^ mice was performed by genotyping using primers for *LacZ*: forward, AATGGTCTGCTGCTGCTGAAC; reverse, GGCTTCATCCACCACATACAG. Mice of varying ages and sexes were used for experiments.

### Western blotting

Cells were treated with vehicle (DMSO), Yoda1, or compound 2e at the appropriate times prior to lysing. Cells were harvested in lysis buffer containing 10 mm Tris (pH 7.5), 150 mm NaCl, 0.5 mm EDTA, 0.5% NP-40, MiniComplete protease inhibitors (Roche), and PhosSTOP phosphatase inhibitors (Roche). A protein quantification assay was then performed using the DC Protein Assay (Bio-Rad). 25 μg of protein was loaded on a 10% polyacrylamide gel. After resolution by electrophoresis, samples were transferred to PVDF membranes, and Western blotting was performed as described previously (38) using a primary antibody for phospho-p38 MAPK (9215, Cell Signaling Technology). Membranes were reprobed with p38α antibody (9228, Cell Signaling Technology) to confirm equal protein loading. Species-appropriate secondary antibodies (GE Healthcare) and ECL Prime Western blotting detection reagent (GE Healthcare) were used for visualization. Syngene G:BOX Chemi XT4 was used for imaging alongside GeneSys image acquisition software for densitometric analysis.

### Intracellular Ca^2+^ measurements

Changes in [Ca^2+^]*_i_* were measured using the ratiometric Ca^2+^ indicator dye Fura-2/AM and a 96-well fluorescence plate reader (FlexStationII384, Molecular Devices) controlled by SOFTmax PRO software v5.4.5. Cardiac fibroblasts and HUVECs were plated in clear 96-well plates (Corning) and HEK T-REx-293 cells in black, clear-bottomed 96-well plates (Grenier) at a confluence of 90% 24 h before experimentation. Cells were incubated for 1 h at 37 °C in standard bath solution (SBS; 130 mm NaCl, 5 mm KCl, 8 mm D-glucose, 10 mm HEPES, 1.2 mm MgCl_2_, and 1.5 mm CaCl_2_ (pH 7.4)) containing 2 μm Fura-2 in the presence of 0.01% pluronic acid (Thermo Fisher Scientific) to aid dispersion. During all Fura-2 assays involving cardiac fibroblasts, 2.5 mm probenecid (Sigma-Aldrich) was present to prevent extrusion of the Fura-2 indicator (39). Cells were washed with SBS and then incubated at room temperature for 30 min, during which time inhibitors were added when applicable. Stimuli were injected at 60 s of recording. The change in intracellular Ca^2+^ concentration (Δ[Ca^2+^]*_i_*) was measured as the ratio of Fura-2 emission (510 nm) intensity at 340 nm and 380 nm.

### Gene silencing

Murine cardiac fibroblasts were grown to 80% confluence and transfected with 10 nm Piezo1-specific Silencer Select Pre-Designed siRNA (4390771, siRNA s107968, Life Technologies) or Silencer Select Negative Control No. 1 siRNA (4390843, Life Technologies) using Lipofectamine RNAiMAX reagent (Life Technologies) in Opti-MEM (Gibco) according to the manufacturer's instructions. Medium was replaced with full-growth medium 24 h later. For human cardiac fibroblasts, cells were grown to 90% confluence and transfected with 20 nm Piezo1-specific Silencer Select Pre-designed siRNA (4392420, siRNA s18891, Thermo Fisher Scientific) or ON-TARGETplus Nontargeting Pool siRNA (D-01810-10-20, Dharmacon) using Lipofectamine 2000 in Opti-MEM according to the manufacturer's instructions. Medium was replaced with full-growth medium after 4.5 h. Cells were used for experimentation 48 h after transfection.

### Patch clamp electrophysiology

Ionic currents were recorded through cell-attached patches using a standard patch clamp technique in voltage clamp mode. Patch pipettes had a resistance of 4–6 megaohm when filled with pipette solution. An ionic solution composed of 145 mm CsCl, 2 mm MgCl_2_, 10 mm HEPES, 5 mm ATP, 0.1 mm GTP, and 1 mm EGTA (titrated to pH 7.2 using CsOH) was used in the pipette. The bath solution was SBS. Recordings were at a constant holding potential of +80 mV (applied to the patch pipette). 200-ms pressure steps were applied to the patch pipette with an interval of 10 s using a high-speed pressure clamp HSPC-1 system (ALA Scientific Instruments). All recordings were made with an Axopatch-200B amplifier (Axon Instruments, Inc.) equipped with Digidata 1550B and pClamp 10.6 software (Molecular Devices) at room temperature. Currents were filtered at 2 kHz and digitally sampled at 20 kHz. Data were analyzed using pClamp 10.6 and the MicroCal Origin 2018 (OriginLab Corp.) software packages. All recordings were made blind (*i.e.* without knowledge of which cells had been transfected with control or Piezo1 siRNA).

### Cell viability assay

Cardiac fibroblasts were plated at 80% confluency and incubated overnight (37 °C, 5% CO_2_). Vehicle (DMSO), Yoda1 (10 μm), or staurosporine (1 μm) were applied the following day, and cells were incubated for 24 h prior to commencing the live/dead cell viability assay (Thermo Fisher Scientific) according to the manufacturer's instructions. Cells were imaged using the IncuCyte ZOOM Live Imaging System (Essen Bioscience). The total number of fluorescent cells in each well was calculated using built-in algorithms, using an average from 9 images/well of a 12-well plate. The mean data are shown as the number of live cells relative to the total number of cells.

### Stretch experiments

Human cardiac fibroblasts were seeded at 1 × 10^5^ cells/well on collagen-coated membranes (BioFlex 6-well culture plates). 72 h after transfection, cells were stretched while in serum-free medium using an FX-4000 or FX-5000 Flexercell Tension System (Flexcell International) to equibiaxially elongate the cell-seeded elastic membrane against a loading post. Elongation at 10% strain and 1 Hz was applied to the cells for 24 h. A 6-well stretching plate was housed in the incubator (37 °C, 5% CO_2_) alongside unstimulated cells adhered to BioFlex plates, which served as static controls. RNA was extracted, and conditioned medium was collected. Quantitative RT-PCR and ELISA were used to quantify gene and protein expression, respectively.

### ELISA

Conditioned media were collected, centrifuged to remove cellular debris, and stored at −20 °C for subsequent analysis. The concentration of IL-6 in the media was measured by ELISA (M6000, R&D Systems) according to the manufacturer's instructions. Samples were diluted 1:5–1:30 prior to analysis.

### Multiplex kinase activity profiling

PamGene serine-threonine kinase (STK) multiplex activity assays were used to investigate STK activity. Murine cardiac fibroblasts were treated with vehicle or Yoda1 for 10 min, collected, and lysed using mammalian protein extraction reagent lysis buffer (Thermo Fisher Scientific) containing Halt phosphatase and Halt protease inhibitor cocktails (Pierce) for 30 min on ice. A protein quantification assay was then performed as stated above, and samples were snap-frozen in liquid nitrogen. 1.2 μg of protein was loaded onto each STK PamChip array. Phosphorylation of PamChip peptides was monitored by PamStation 12 following the manufacturer's protocols as described previously (40). Signal intensities were analyzed using PamGene's BioNavigator software as Yoda1-treated *versus* DMSO treatment after 10 min. Permutation analysis resulted in a specificity score (mapping of peptides to kinases) and a significance score (difference between treatment groups) for each kinase. The combined score was used to rank and predict top kinase hits.

### Statistical analysis

OriginPro 2015 (OriginLab) and GraphPad Prism version 7.05 (GraphPad Software) were used for data analysis, and OriginPro 2015 was used to prepare the charts and graphs. Averaged data are presented as mean ± S.E., where *n* represents the number of independent experiments and *N* indicates the total number of replicates within the independent experiments. Data were log-transformed prior to statistical analysis. For comparisons between two sets of data, paired or unpaired Student's *t* tests were used as appropriate. For multiple comparisons with a single factor, one-way ANOVA was used (*p* values in the figure legends) with Tukey post hoc test (*p* values in the figures). For analysis of two factors, regular or repeated measures two-way ANOVA was used (*p* values in the figure legends) with Sidak post hoc test (*p* values in the figures). *p* < 0.05 was considered statistically significant. For IC_50_ determination, data were normalized to vehicle, and curves were fitted using the Hill equation.

## Author contributions

N. M. B., K. M., and V. S. data curation; N. M. B., M. J. L., V. S., H. T. G., J. S., J. L., F. A. v. N., and K. E. P. methodology; N. M. B. and N. A. T. writing-original draft; N. M. B., K. M., F. A. v. N., D. J. B., and N. A. T. writing-review and editing; M. J. L., H. T. G., E. L. E., K. C., R. F., J. S., J. L., F. A. v. N., K. E. P., D. J. B., and N. A. T. resources; J. L., M. J. D., F. A. v. N., D. J. B., and N. A. T. supervision; J. L., M. J. D., D. J. B., and N. A. T. funding acquisition; D. J. B. and N. A. T. conceptualization; N. A. T. project administration.

## Supplementary Material

Supporting Information

## Footnotes

5 The abbreviations used are:

ECM: extracellular matrix
MAPK: mitogen-activated protein kinase
HUVEC: human umbilical vein endothelial cell
MACS: magnetic antibody cell separation
Het: heterozygous
ANOVA: analysis of variance
ERK: extracellular signal-regulated kinase
CDK: cyclin-dependent kinase
JNK: c-Jun N-terminal kinase
SBS: standard bath solution
STK: serine-threonine kinase
CF: cardiac fibroblast(s).
