# Supplementary material for: Mechanically activated Piezo1 channels of cardiac fibroblasts stimulate p38 mitogen-activated protein kinase activity and interleukin-6 secretion
Source: J Biol Chem. 2019 Oct 4;294(46):17395–408. doi: 10.1074/jbc.RA119.009167 (PMC6873183; doi:10.1074/jbc.RA119.009167)
Supplement: Supporting Information [file supp_294_46_17395__index.html]

Mechanically activated Piezo1 channels of cardiac fibroblasts stimulate p38 mitogen-activated protein kinase activity and interleukin-6 secretion — Cardiac fibroblast Piezo1 activation induces IL-6 secretion — Mechanically activated Piezo1 channels of cardiac fibroblasts stimulate p38 mitogen-activated protein kinase activity and interleukin-6 secretion — Cardiac fibroblast Piezo1 activation induces IL-6 secretion — Supporting Information 

# Mechanically activated Piezo1 channels of cardiac fibroblasts stimulate p38 mitogen-activated protein kinase activity and interleukin-6 secretion

## Supporting Information

- Supporting Information (to be published online) - Supplemental Figs S1 and S2
- Supporting Information - Table S1 - Table S1
