# Supplementary material for: Mechanically activated Piezo1 channels of cardiac fibroblasts stimulate p38 mitogen-activated protein kinase activity and interleukin-6 secretion
Source: J Biol Chem. 2019 Oct 4;294(46):17395–408. doi: 10.1074/jbc.RA119.009167 (PMC6873183; doi:10.1074/jbc.RA119.009167)
Supplement: Supporting Information [file supp_RA119.009167_152571_2_supp_404610_pyr5x9.docx]

**Mechanically-activated Piezo1 channels of cardiac fibroblasts stimulate p38 mitogen-activated protein kinase and interleukin-6 secretion**

Nicola M. Blythe, Katsuhiko Muraki, Melanie J. Ludlow, Vasili Stylianidis, Hamish T. J. Gilbert, Elizabeth L. Evans, Kevin Cuthbertson, Richard Foster, Joe Swift, Jing Li, Mark J. Drinkhill, Frans A. van Nieuwenhoven, Karen E. Porter, David J. Beech and Neil A. Turner

**SUPPORTING INFORMATION**

**Figure S1.** Inhibition of Yoda1-evoked Ca^2+^ entry in human cardiac fibroblasts.

**Figure S2.** Yoda1, but not compound 2e, induces Ca^2+^ entry and IL-6 expression in human cardiac fibroblasts.

**Table S1.** PamChip multiplex kinase activity profiling of Yoda1-induced serine/threonine kinase activity. [Excel file]

**Figure S1. Inhibition of Yoda1-evoked Ca^2+^ entry in human cardiac fibroblasts.** Representative intracellular Ca^2+^ traces and mean data after human cardiac fibroblasts were exposed to 10 μM gadolinium (Gd^3+^), 30 μM ruthenium red (RuR), 10 μM Dooku1 or vehicle for 30 min before activation of Piezo1 by application of 2 μM Yoda1. Data was normalized to vehicle-treated cells. Repeated measures 1-way ANOVA: P<0.0001, F=413.2 (n/N=3/9). Post hoc test: ***P<0.001 versus vehicle-treated cells.

**Figure S2. Yoda1, but not compound 2e, induces Ca^2+^ entry and IL-6 expression in human cardiac fibroblasts.** Representative Ca^2+^ traces illustrating Ca^2+^ entry evoked by 10 μM Yoda1 or 10 µM compound 2e in **(A)** murine cardiac fibroblasts, **(B)** human cardiac fibroblasts or **(C)** HEK T-REx-293 cells heterologously expressing mouse Piezo1 (n/N=3/9). Human cardiac fibroblasts transfected with scrambled or Piezo1-specific siRNA were exposed to vehicle, 10 µM Yoda1 or compound 2e for 6 h before measuring mRNA levels by RT-PCR with primers for **(D)** *PIEZO1* or **(E)** *IL6* (n=3). Expression is measured as % of housekeeping control, *GAPDH*. **P<0.01 versus control siRNA (paired t test), *P<0.05 versus vehicle control siRNA (repeated measures 2-way ANOVA; compound P=0.0883, siRNA P=0.8567). All other changes not statistically significant.
